# Pharmacognostic Evaluation and HPLC–PDA and HS–SPME/GC–MS Metabolomic Profiling of *Eleutherococcus senticosus* Fruits

**DOI:** 10.3390/molecules26071969

**Published:** 2021-03-31

**Authors:** Filip Graczyk, Maciej Strzemski, Maciej Balcerek, Weronika Kozłowska, Barbara Mazurek, Michał Karakuła, Ireneusz Sowa, Aneta A. Ptaszyńska, Daniel Załuski

**Affiliations:** 1 Department of Pharmaceutical Botany and Pharmacognosy, Ludwik Rydygier Collegium Medicum, Nicolaus Copernicus University, Marie Curie-Skłodowska 9, 85-094 Bydgoszcz, Poland; balcerek@cm.umk.pl (M.B.); daniel_zaluski@onet.eu (D.Z.); 2 Department of Analytical Chemistry, Medical University of Lublin, Chodźki 4a, 20-093 Lublin, Poland; maciej.strzemski@poczta.onet.pl (M.S.); michal.karakula@umlub.pl (M.K.); i.sowa@umlub.pl (I.S.); 3 Department of Pharmaceutical Biology, Wroclaw Medical University, Borowska 211, 50-556 Wroclaw, Poland; weronika.kozlowska@umed.wroc.pl; 4 Analytical Department, New Chemical Syntheses Institute, Aleja Tysiąclecia Państwa Polskiego 13a, 24-110 Puławy, Poland; barbara.mazurek@ins.lukasiewicz.gov.pl; 5 Department of Immunobiology, Institute of Biological Sciences, Faculty of Biology and Biotechnology, Maria Curie-Skłodowska University, Akademicka 19 Str., 20-033 Lublin, Poland; anetaptas@wp.pl

**Keywords:** *Eleutherococcus senticosus*, fruits, eleutherosides, nutri-pharmacological, metabolomics, herbs

## Abstract

*Eleutherococcus senticosus* (Rupr. et Maxim.) Maxim. is a medicinal plant used in Traditional Chinese Medicine (TCM) for thousands of years. However, due to the overexploitation, this species is considered to be endangered and is included in the Red List, e.g., in the Republic of Korea. Therefore, a new source of this important plant in Europe is needed. The aim of this study was to develop pharmacognostic and phytochemical parameters of the fruits. The content of polyphenols (eleutherosides B, E, E1) and phenolic acids in the different parts of the fruits, as well as tocopherols, fatty acids in the oil, and volatile constituents were studied by the means of chromatographic techniques [HPLC with Photodiode-Array Detection (PDA), headspace solid-phase microextraction coupled to gas chromatography-mass spectrometry (HS–SPME/GC–MS)]. To the best of our knowledge, no information is available on the content of eleutherosides and phenolic acids in the pericarp and seeds. The highest sum of eleutheroside B and E was detected in the whole fruits (1.4 mg/g), next in the pericarp (1.23 mg/g) and the seeds (0.85 mg/g). Amongst chlorogenic acid derivatives (3-CQA, 4-CQA, 5-CQA), 3-CQA was predominant in the whole fruits (1.08 mg/g), next in the pericarp (0.66 mg/g), and the seeds (0.076 mg/g). The oil was rich in linoleic acid (C18:3 (n-3), 18.24%), ursolic acid (35.72 mg/g), and α-tocopherol (8.36 mg/g). The presence of druses and yellow oil droplets in the inner zone of the mesocarp and chromoplasts in the outer zone can be used as anatomical markers. These studies provide a phytochemical proof for accumulation of polyphenols mainly in the pericarp, and these structures may be taken into consideration as their source subjected to extraction to obtain polyphenol-rich extracts.

## 1. Introduction

Plant-based metabolites have served as lead compounds for many important drugs, such as morphine, digoxin, quinine, hyoscyamine, salicylic acid, and artemisinin [1]. According to the WHO (World Health Organization), about 80% of the world’s population use plants in the primary health system, both in the developing and developed countries. For instance, 14% of the Russian population use them regularly and 44% from time-to-time [2]. 

One of the best-known plants, used as a source of pharmacologically active and nutritional molecules, is *Eleutherococcus senticosus* (Siberian ginseng). The plant is native to Russia, the Far East, China, Korea, and Japan. The fruits of *E. senticosus* have been used in Russia for many years as a tonic on the central nervous system and as an adaptogen. Modern research is focused on elucidation of the pharmacological activities of wild fruits, including their antioxidant, antimicrobial, and anticancer effects. An acidified 80% methanol extract showed high xanthine oxidase and AChE inhibitory activities. The anticancer activity of the extract was also proven by screening various human cell lines, including LNCaP (prostate cells), MOLT-4F (leukemia cells), A549 (lung cells), ACHN (renal cells) HCT-15 (colon cells), and SW-620 (colon cells). The latest reports indicate their immunostimulatory and anti-inflammatory activities and an increase in the number of leukocytes. The intractum from the fruits was found to stimulate human leukocyte resistance to the VSV (*Vesicular Stomatitis Virus*) infection via reduction of viral replication, which might be associated with increased secretion of interleukin 10 (IL-10). Besides medical applications, the fruits are industrially used in the production of phyto-jam (0.8–1.6 kg/100 kg of the product). Considering the data mentioned above, the consumption of wild edible fruits of *E. senticosus* by local communities in many developing countries is gaining increasing interest [3,4,5,6,7]. 

According to the European Medicines Agency (EMA), the pharmacological activity of *E. senticosus* in traditional applications is attributed to secondary metabolites called eleutherosides. They are very varied with saponins, lignans, coumarins, and phenylpropanoids as their aglycons. Eleutheroside B (syringin 4-β-d-glucoside) and E ((−)-syringaresinol 4,4″-*O*-β-d-diglucoside) are the major compounds and, according to the European Pharmacopoeia, their sum should not be less than 0.08%. Eleutherosides are thought to be the most active compounds present mainly in the roots. Although the fruits are used as ingredients in the production of galenic formulations, the roots are a major subject of research while the fruits remain largely unknown. The fruits are rich in flavonoids (13.4–14.4 mg/g ext.), polyphenols (38.5–41.1 mg/g ext.), minerals (Ca 3730–4495, Mg 1430–1540, Fe 35.4–53, Mn 75.2–88.3, Zn 18.9–41.0, Cu 3.34–13, Se 0.19–061; mg/kg respectively), and essential oil (0.3%, v/d.w.). Interestingly, a large amount of *myo*-inositol and d-mannitol was found as well (267.5 and 492.5 mg/g dry extract, respectively) [8,9,10].

**Figure 1:**
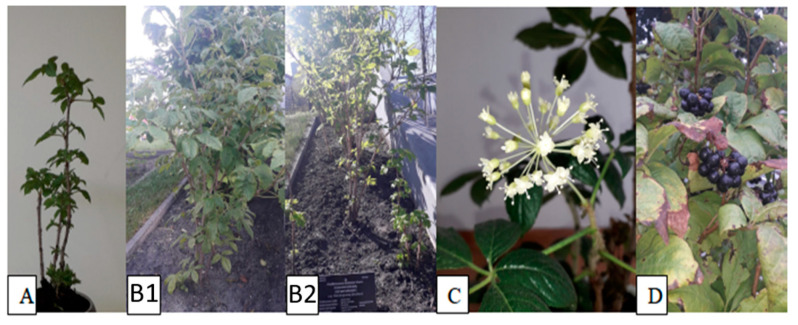
Morphological characteristics of the investigated species; (**A**) 1-year-old plant, general morphology; (**B1**,**B2**) 4-year-old plants growing in the crop field, general morphology; (**C**) flowers; (**D**) mature fruits.

As demonstrated by previous studies conducted by Załuski, *E. senticosus* is successfully cultivated in the botanical garden in Rogów (Central Polish Lowlands), and some methods required for determination of phenolic metabolites in the plant material and the biological activity of extracts have been developed [11,12]. The observations have also been confirmed by Bączek, who studied the impact of growth conditions on accumulation of biologically active compounds in organs of two-, three-, and four-year-old plants [13]. Nevertheless, the species cultivated in Bydgoszcz (North Polish Lowlands) has not yet been studied in detail (Figure 1). Preliminary information on chemical compounds in the *E. senticosus* fruits intractum have been previously reported by Graczyk et al. [8]. To the best of our knowledge, no data are available on the localization of eleutherosides and phenolic acids in anatomical structures or on pharmacognostic features that can be used as markers in quality assessment. It is well known that phytochemicals can be accumulated in different fruit parts, e.g., *Citrus limon* Burm. contains the highest amount of chlorogenic or caffeic acids or hesperidin in the peel [14,15,16,17]. In addition, there is no information about the oil in the fruits, which may be of interest e.g., for the food industry [10,18,19,20]. 

To confirm our hypothesis, this study was focused on the quantitative analysis of phytochemicals with their localization in the anatomical fruit parts (seeds, pericarp) and on anatomical features that can be possibly used in the quality control of this pharmaceutically important plant.

## 2. Results and Discussion

### 2.1. Microscopic Pharmacognostic Features of Fruits

The quality control of plant-based material is important to ensure the best quality of products. For many years, plant materials imported to Europe have been of bad quality (adulterated or substituted) [21]; therefore, to ensure good quality, new protocols for a new herbal material are needed. The fruits of *Eleutherococcus senticosus* species growing in Asia and cultivated in Poland or other European countries have not yet been evaluated in terms of pharmacognostic features. The poor-quality plant material now offered on the market necessitates development of proper well-controlled production procedures. The development of a pharmacognostic protocol including microscopic and phytochemical studies will help in identification of these fruits and protect from adulteration.

**Figure 2:**
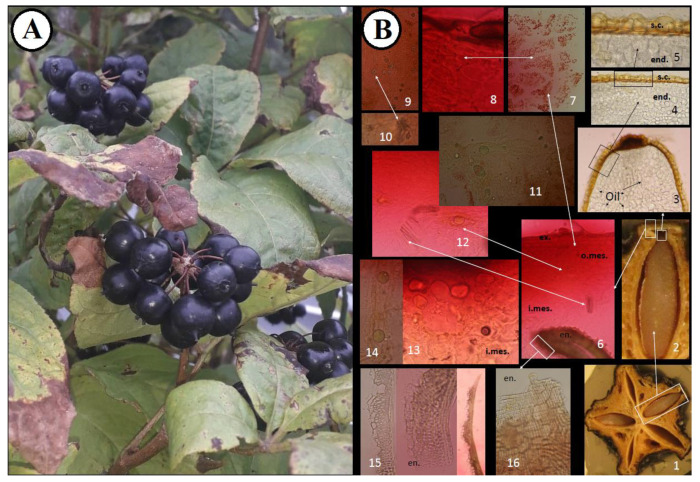
(**A**). Morphological structure and anatomical features of *E. senticosus* fruits. (**B**). **1**. Transverse section of the fruit, **2**–**3**. Seed, **4**–**5**. Seed coat and endosperm, **6**. Pericarp, **7**–**8**. Outer zone of mesocarp, **9**–**10**. Mesocarp with druses, **11**–**13**. Inner zone of mesocarp with secretory canals and oil droplets (transverse section), **14**. Inner zone of mesocarp with secretory canals and oil droplets (longitudinal section), **15**. Endocarp (transverse section), **16**. Endocarp (tangential section). end.—endosperm, s.c.—seed coat, en.—endocarp, i.mes.—inner zone of mesocarp, o.mes.—outer zone of mesocarp, ex.—exocarp.

**Figure 3:**
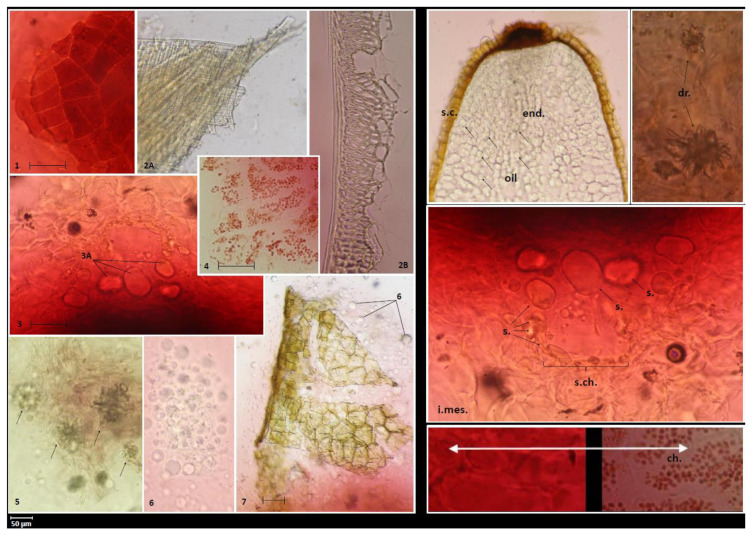
Diagnostic features of powdered fruits. **1**. Polygonal cells of fruit epidermis (exocarp). **2**. Inner layers of endocarp cells are represented by fibers, oriented askew to stone axis (2A—tangential section, 2B—transverse section). **3**. Secretory canal (cross-section) in mesocarp, parenchymal cells (3A—visible oil content). **4**. Outer part of the mesocarp composed of parenchymal cells containing red plastids (chromoplasts). **5**. Druses in mesocarp cells located closer to the endocarp. **6**. Drops of fatty oil from the endosperm. **7**. Seed coat built of large cells with brown contents. Diagnostic features of the fruits. s.c.—seed coat, end.—endosperm, i.mes.—inner zone of mesocarp, s.ch.—secretory canals, s.—secretion (essential oil), dr.—druses, ch.—chromoplasts.

The characteristic anatomical features of the fruits are shown in Figure 2 and Figure 3. The anatomical structure of the *E. senticosus* fruits is characteristic for most species from the Araliaceae family. The microscopic studies of the transverse section showed the presence of secretory canals filled with yellow oil droplets in the inner zone of the mesocarp. The presence of druses and yellow oil droplets in the inner zone of the mesocarp and red chromoplasts in the outer zone of the mesocarp visible in the transverse fruit section are distinguishing features that can be used as anatomical markers. This study is a complement of the research conducted by Solomonowa et al. [20], in which the morphological-anatomical and morphometric study of fruits were carried out. The morphometric indicators of the fruits are as follows: fruit length 9.95 mm, fruit diameter 4.65, stone length 5.25 mm, and stone width 1.98 mm.

The powder microscopy of the fruits (Figure 3) shows epidermis cells tightly adjacent to each other. The endocarp contains fibers oriented askew to the stone axis. The secretory canals in the mesocarp are filled with oil, probably essential oil, rounded parenchymatous cells. In turn, the druses and chromoplasts in the mesocarp and fatty oil drops in the endosperm can be considered as diagnostic features. The parameters analyzed here are useful for identification and authentication of this medicinally important plant and will provide the latest knowledge for the development of herbal monographs as recommended by the European Medicines Agency.

### 2.2. HPLC–PDA and GC–MS Metabolite Profiling of the Fruits

#### 2.2.1. HPLC–PDA Analysis of Eleutherosides B, E, and E1 in the Anatomical Structures of the Fruits

For the analysis of the metabolome, such approaches as HPLC, TLC–UV, GC–MS, LC-MS, MS^n^, and NMR-spectrometry are currently used. TLC coupled with densitometry and HPLC or HPLC–MS have usually been applied to analyze eleutherosides. High-performance liquid chromatography (HPLC) is a leading technique for detection of plant-based metabolites and is very often used as a pilot technique for separation and identification of phytochemicals [10,11,12].

**Figure 4:**
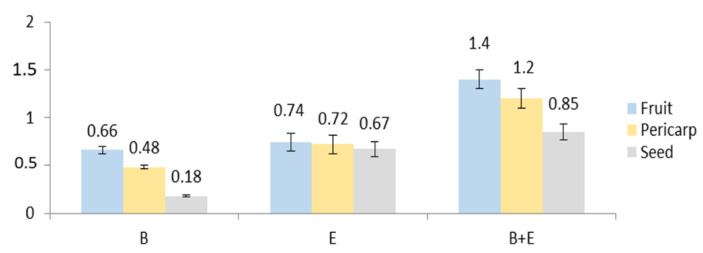
Contents of eleutherosides (mg/g extract): B—eleutheroside B, E—eleutheroside E, B + E—sum of eleutheroside B and eleutheroside E.

**Figure 5:**
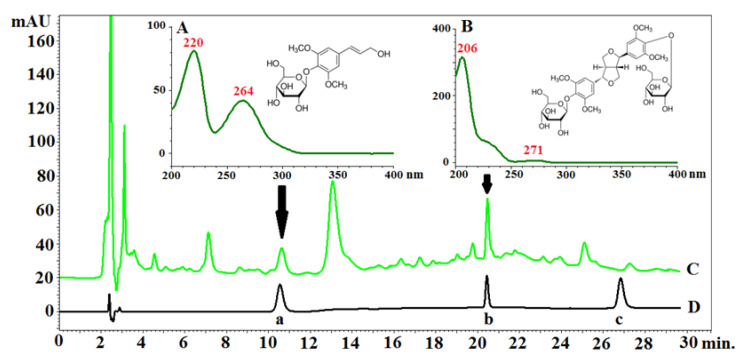
Chromatogram of *Eleutherococcus senticosus* fruit and reference standards with PDA spectra and structures of determined compounds: (**A**)—PDA spectrum and chemical structure of eleutheroside B; (**B**)—PDA spectrum and chemical structure of eleutheroside E; (**C**)—chromatogram of the extract of *Eleutherococcus senticosus* fruit; (**D**)—chromatogram of the mixture of standards (**a**—eleutheroside B, **b**—eleutheroside E, **c**—eleutheroside E1). RP18e LiChrospher 100 column (Merck, Darmstadt, Germany) (25 cm × 4.0 mm i.d., 5 µm particle size) at 25 °C; mixtures of water (solvent A) acetonitrile (solvent B) and both acidified with 0.025% of trifluoroacetic acid were used as the mobile phase. The compounds were separated by gradient elution with the following program: 0.0–8.0 min A 90%, B 10%; 8.1–18.0 min A 90–80%; B 10–20%, 18.1–30.0 min A 80%, B 20%. The flow rate was 1.0 mL/min.

The extraction of the fruits, seeds, and pericarp obtained from the 4-year-old plant resulted in 20, 26, and 33% dry extract yield, respectively. No data on the characterization of the anatomical parts of *E. senticosus* fruits and the distribution of their active constituents have been published before. Plant metabolites exhibit a very broad range of polarities. This means that only part of the plant’s chemodiversity is present in any plant extract. Among the three studied eleutherosides (eleutheroside B, E, and E1), only eleutheroside B and E have been found in the largest amount in the whole fruits i.e., 0.66 and 0.74 mg/g d. ext., respectively (Figure 4 and Figure 5). Considering the distribution of these compounds, a higher quantity was noticed in the pericarp than in the seeds; however, there was no significant difference between the pericarp and seeds, especially in the case of eleutheroside E. The latest studies conducted by Graczyk et al. [8] have revealed the absence of eleutherosides in the intractum made of the fresh fruits. This may result from their medium polarity. Eleutherosides are usually well extracted with 75% (*v/v*) methanol or ethanol; therefore, we suppose that the ethanol concentration (40% *v/v*) used to prepare the intractum may have been too low to extract eleutherosides. Moreover, the extraction of the whole fruits also means that some compounds will not be extracted, and the intractum was prepared through maceration, while ultrasounds were applied in this study.

Bączek [13] showed that the content of eleutherosides B and E in methanol fruit extract obtained from a 4-year-old plant was 35.6 and 29.7 (mg/100g), respectively. It should be mentioned that eleutherosides were not present in the fruits of 2- and 3-year-old plants, respectively. Unfortunately, the authors did not provide information about the calculation, i.e., it was not specified whether 100 g referred to the plant material or the extract. This makes the comparison difficult and, in many cases, unreliable. Taking into consideration the sum of eleutheroside B and E, the extract from the whole fruits contains a higher quantity of eleutherosides. However, it is very important that the pericarp contains more eleutherosides than the seeds, which means that the seeds may be used in the micropropagation of the species. *Eleutherococcus senticosus* is included in the Red List in some countries [8]; in this case, the pericarp may serve as a source of eleutherosides, while the seed (embryo) may be used for in vitro germination to develop seedlings and increase the number of botanical specimens.

#### 2.2.2. HPLC–PDA Analysis of Phenolic Acids in the Anatomical Structures of the Fruits

**Figure 6:**
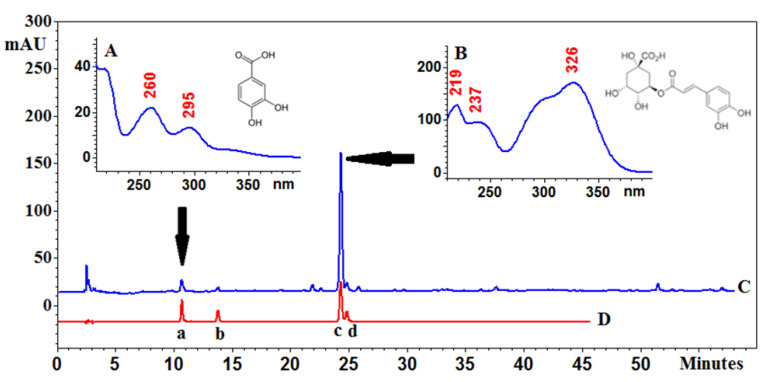
Chromatogram of *Eleutherococcus senticosus* fruits (**C**) and a mixture of reference standards (**D**): a—protocatechuic acid, b—neochlorogenic acid, c—chlorogenic acid and d—cryptochlorogenic acid with PDA spectra and structures of the main compounds: (**A**)—protocatechuic acid and (**B**)—chlorogenic acid. C18 core-shell column (Kinetex, Phenomenex, Aschaffenburg, Germany) (25 cm × 4.6mm i.d., 5 μm particle size) at 25 °C, a mixture of water (solvent A) and acetonitrile (solvent B) with 0.025% of trifluoroacetic acid; gradient elution program: 0.0–5.0 min A 95%, B 5%; 5–60 min A from 5 to 20%, and B from 95–80%. The flow rate was 1.0 mL/min.

Phenolic acids play a vital function as non-nutritional constituents of human diet and pharmacologically active compounds. In some fruits, they are accumulated in different parts. In this study, it has been found that the whole fruits contain a high amount of chlorogenic acid; 3-CQA (1.08 mg/g d. ext.), in comparison to the pericarp (0.66 mg/g d. ext.) and seeds (00.76 mg/g d. ext.) (Table 1, Figure 6).

Chlorogenic acid is the main phenolic component in coffee and beverages prepared from herbs, fruits, and vegetables. The results presented in literature revealed that the content of chlorogenic acid in methanol extracts of fruits from a 4-year-old plant was 409.2 mg/100g [13]. No chlorogenic acid has been detected in the fruits of 2- and 3-year-old plants, respectively. Other studies have shown that the water extract from the fruits of *E. senticosus* contained 0.57 mg/g dry weight of chlorogenic acid [9].

### 2.3. HS–SPME/GC–MS Analysis of Volatile Metabolites in the Fruits

**Figure 7:**
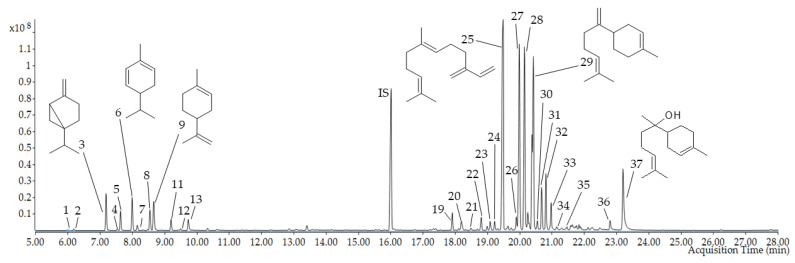
HS–SPME/GC–MS chromatogram of volatile compounds in *E. senticosus* fruits. The numbers correspond to the compounds in Table 2; IS—internal standard.

HS–SPME coupled with the GC–MS analysis identified 38 volatile constituents (VOCs) in the *E. senticosus* fruits, representing 93.9% of the total number. The detected compounds belong to several classes, such as monoterpene hydrocarbons and oxygenated monoterpenes (13.2%) and sesquiterpene hydrocarbons and oxygenated sesquiterpenes (76.6%). The percentage composition of the identified compounds is listed in the elution order in Table 2 and Figure 7 and Figure S2. (E)-β-farnesene (19.46 ± 0.78%), germacrene d (12.88 ± 1.10%), (E,Z)-*α*-farnesene (12.84 ± 0.85%), and *β*-bisabolene (12.64 ± 0.70%) were the main compounds identified in the *E. senticosus* fruits.

Previous reports on the essential oil from *E. senticosus* fruits indicated β-caryophyllene and humulene [7] or spathulenol [10] as the main compounds. However, the results of the present study revealed farnesene and bisabolene-type derivatives as dominant compounds showing more similarity to the essential oil from leaves described by Zhai et al. [22]. Studies conducted on *E. senticosus* root material obtained from different regions (Russia, China—two different sources) showed significant fluctuations in the quantity of the compounds, which may be connected with differentiation of the chemotypes [23]. Moreover, Załuski and Janeczko reported differences in the composition of fruit essential oil connected with duration of storage [10].

### 2.4. GC–MS and HPLC–PDA Profiling of the Fruit Fatty Oil

**Figure 8:**
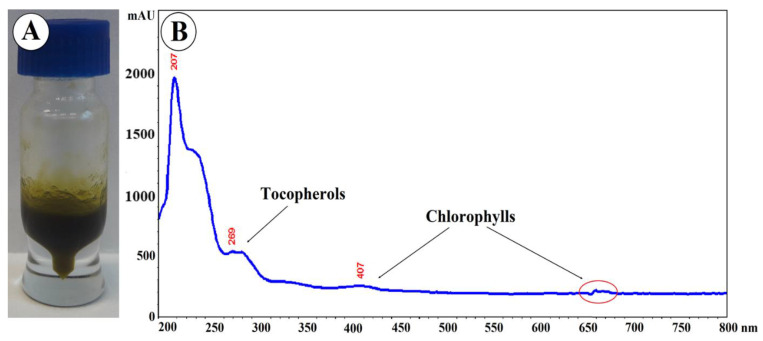
(**A**) Photography and (**B**) UV–VIS spectroscopic fingerprint of *Eleutherococcus senticosus* fruit oil.

The oil obtained by hexane extraction of ground fruits constituted 5.4 ± 0.015% of their weight. The oil is a semi-solid, yellow-green substance with a characteristic aroma (Figure 8A) absorbing UV–VIS radiation in the range of 190–430 nm and at a wavelength of 660–680 nm (Figure 8B). Absorbance at a wavelength of 269 nm may indicate the presence of tocopherols, especially α-tocopherol absorbing radiation in the range of 265–310 nm [24]. Absorbance at 407 and 660–680 nm may indicate the presence of chlorophylls [25]. It was shown that the content of chlorophyll A and chlorophyll B was 12.43 mg/g and 3.67 mg/g, respectively, and the content of carotenoids in the tested oil was 10.13 mg/g.

#### 2.4.1. GC–MS Analysis of Fatty Acids

**Figure 9:**
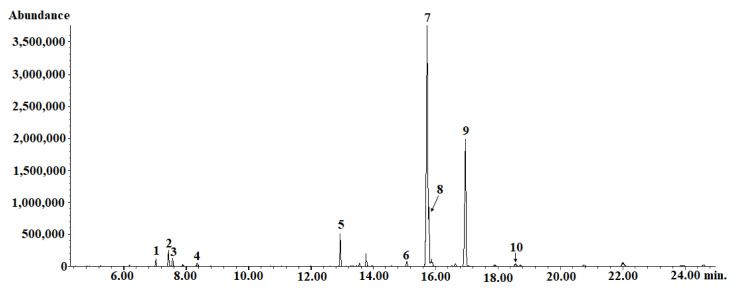
GC–MS chromatogram of *Eleutherococcus senticosus* fruit oil. 1-(Z)-β-farnesene; 2-α-bergamotene; 3-α-farnesene; 4-β-cubebene; 5-methyl palmitate; 6-methyl stearate; 7-trimethylsulfonium ursolate; 8-methyl oleate; 9-methyl linoleate; 10-methyl linolenate. HP-88 Agilent 45 capillary column (60 m × 0.25 mm; 0.20 μm film thickness). The oven temperature was programmed from 110 °C to 190 °C with 8 °C/min held for 2 min at 110 °C and 13 min at 190 °C. The temperature of the injector was 250 °C. Helium was used as a carrier gas at a flow rate of 1.2 mL/min A quadrupole mass spectrometer with electron ionization (EI) at 70 eV and with a full scan type acquisition mode (50 *m/z* to 500 *m/z*) was used as a detector connected with GC. The temperature of the MS source and the MS quadrupole was set to 230 °C and 150 °C, respectively.

The analysis of the oil by gas chromatography with mass spectrometry showed the presence of five fatty acids, including three unsaturated acids (linoleic, oleic, and α-linolenic acids), the total content of which was 24% (Table 3). The dominant fatty acid was linoleic acid (over 18%), while the content of α-linolenic acid was only 0.4%. The content of saturated acids was only 2.5%. Thus, the *E. senticosus* fruit oil is a rich source of unsaturated acids, but the proportion of n-3 acids to n-6 acids is not beneficial for human health [26]. In addition, the analysis showed the presence of four components of the essential oil and trimethylsulfonium ursolate—a derivative of ursolic acid and TMSH. An example of a chromatogram is presented in Figure 9, and the mass spectrum for fatty acids is shown in Figure S1.

The chromatographic analysis (HPLC–PDA) revealed a dominant peak with a retention time of 7.25 min. This peak was identified as a mixture of β- and γ-tocopherols, and their content was determined to be 1.36 mg/g oil. The α- and δ-tocopherol content was 0.29 and 0.33 mg/g of oil, respectively (Table 4). Grilo et al. assessed the content of tocopherols in commonly used oils (rapeseed, sunflower, corn, and soybean). The content of α-tocopherol ranged from 0.071 to 0.43 mg/g and γ-tocopherol ranged from 0.092 to 0.27 mg/g for soybean and sunflower oils, respectively [27]. Thus, it can be concluded that the *E. senticosus* seed oil contains a moderate amount of α-tocopherol. On the other hand, the tested oil may be a valuable source of β- and γ-tocopherols; however, the assessment of the mutual quantitative relationships of these two compounds requires further research and other methods. Ergönül and Köseoğlu have shown that the content of δ-tocopherol in unrefined soybean oil is 0.12 mg/g, while in rapeseed oil it is 0.012 mg/g [28]. Thus, it can be concluded that the *E. senticosus* fruit oil is quite a rich source of δ-tocopherol, as it contains nearly three-fold higher amounts of this compound than soybean oil.

#### 2.4.2. HPLC–PDA Analysis of Ursolic Acid 

Pentacyclic triterpenes are lipophilic compounds present in the oil fraction [29]. As shown in literature, *E. senticosus* fruits contain ursolic acid [7]. Therefore, the content of this triterpene in *E. senticosus* seed oil was evaluated in the present study. Its content was shown to be 35.72 mg/g of oil (Table 4). Given the oil content in the fruits, it can be concluded that the ursolic acid content in the fruits is over 1.9 mg/g. Our research shows that *E. senticosus* fruits are a much richer source of ursolic acid than previously thought. Jang et al. found that 100 g of fruits contain about 3.5 mg of ursolic acid [7]. The results obtained by Yang’s team were on lower possibly due to the use of methanol in fruit extraction. 

## 3. Materials and Methods

### 3.1. Chemicals and Reagents

The standards of eleutheroside B (≥98.0%), eleutheroside E (≥98.0%), eleutheroside E1 (≥98.0%), protocatechuic acid (≥97%), ursolic acid (≥90%), δ-tocopherol, (+)-γ-tocopherol, (±)-α-tocopherol (analytical standards), methyl palmitate (≥99.0%), methyl stearate (≥99.5%), methyl oleate (≥99.0%), methyl linoleate (≥98.5%), methyl linolenate (≥99.0%), 2-propanol (99.9%), hexane (≥95%), phosphoric acid (≥85%), trimethylsulfonium hydroxide (TMSH) (0.25 M methanolic solution), gradient grade acetonitrile, and trifluoroacetic acid (≥99%) were obtained from Sigma-Aldrich (St. Louis, MO, USA). LC grade methanol (MeOH) was purchased from J.T. Baker (Phillipsburg, NJ, USA). Water for HPLC was purified by ULTRAPURE Millipore Direct-Q^®^ 3UV-R (Merck Millipore, Billerica, MA, USA). Tert-butyl methyl ether (TBME) (99.8%) was purchased from Avantor Performance Materials Poland S.A. (Gliwice, Poland). All other reagents were of analytical grade. Eleutheroside standards were dissolved in 75% methanol (final concentration of 0.16, 0.16, and 0.36 mg/mL for eleutheroside B, E, and E1, respectively). Phenolic acid standards and ursolic acid were dissolved in methanol (final concentration of 0.24, 1.04, 0.23, 0.30, and 0.50 mg/mL for neochlorogenic, chlorogenic, cryptochlorogenic, protocatechuic, and ursolic acid, respectively). Standard solutions were prepared by dilution of stock solutions to appropriate concentrations.

### 3.2. Plant Material and Preparation of the Extract

Mature fruits of 4-year-old *Eleutherococcus senticosus* were collected in the Garden of Medicinal and Cosmetic Plants in Bydgoszcz (Poland) in September 2017 (N: 53°07′36.55″ E: 18°01′51.64″). The plant sample was deposited at the Department of Pharmaceutical Botany and Pharmacognosy, Collegium Medicum, Bydgoszcz, Poland, Cat. Nr. ES 10/2018. Air-dried and powdered fruits (10 g each) were soaked in 100 mL of 75% ethanol for 24 h. Next, the samples were subjected to triple UAE-type extraction (ultrasonic bath—Polsonic, Warsaw, Poland) using 100 and 2 × 50 mL of 75% ethanol. The extraction was performed at room temperature for 15 min for each cycle. Finally, 200 mL of each extract was obtained. After that, the extract was filtered through Whatman no. 4 filter paper. The solvent was dried with an evaporator in vacuum conditions at 45 °C, frozen at −20 °C, and subjected to lyophilization. The dried residue was stored in an exsiccator at 4 °C. The extraction yield was calculated based on the dry weight of the extract [%]. The same steps were made for extraction of the pericarp and seeds.

### 3.3. Microscopic Analysis

The anatomical structure of the fruits was examined microscopically using a compound microscope coupled with a camera, evaluated, and photographed (40X). Chloral hydrate was used as a reagent. Computer images were captured using software ProgRes CapturePro 2.8—Jenoptik optical system.

### 3.4. HPLC–PDA Analysis of Eleutherosides B, E, and E1

The analyses were performed on an EliteLaChrom chromatograph with a PDA detector and EZChrom Elite software (Merck, Darmstadt, Germany). The following chromatographic system was used: an RP18e LiChrospher 100 column (Merck, Darmstadt, Germany) (25 cm × 4.0 mm i.d., 5 µm particle size) at 25 °C; mixtures of water (solvent A) and acetonitrile (solvent B) both acidified with 0.025% of trifluoroacetic acid were used as the mobile phase. The compounds were separated by gradient elution with the following program: 0.0–8.0 min A 90%, B 10%; 8.1–18.0 min A 90–80%, B 10–20%; 18.1–30.0 min A 80%; B 20%; 30.1–45.0 min A 0%, B 100%; 45.1–60.0 min A 90%, B 10%. The flow rate was 1.0 mL/min. Data were collected between 190 and 400 nm. The identity of compounds was established by comparison of retention times and UV spectra with the corresponding standards. Quantitative analysis was performed at λ = 264 nm for eleutheroside B and λ = 206 nm for eleutheroside E and E1. The chromatographic parameters and calibration data for quantification of the investigated eleutherosides are provided in Table S1. 

### 3.5. HPLC–PDA Analysis of Phenolic Acids

The analyses were performed on an EliteLaChrom chromatograph with a PDA detector and EZChrom Elite software (Merck, Darmstadt, Germany). The chromatographic system was as follows: a C18 reversed phase core-shell column (Kinetex, Phenomenex, Aschaffenburg, Germany) (25 cm × 4.6 mm i.d., 5 μm particle size), a mixture of water (solvent A) and acetonitrile (solvent B) with 0.025% of trifluoroacetic acid, and the following gradient elution program: 0.0–5.0 min A 95%, B 5%; 5–60 min A from 5 to 20% and B from 95–80%. The flow rate of the mobile phase was 1.0 mL/min and the temperature of thermostat was set at 25 °C. Data were collected between 210 and 400 nm. The identity of compounds was established by comparison of retention times and UV spectra with the corresponding standards. Quantitative analysis was performed at λ = 326 nm for chlorogenic acids and λ = 260 nm for protocatechuic acid. The chromatographic parameters and calibration data for quantification of the investigated phenolic acids are provided in Table S1.

### 3.6. HS–SPME/GC–MS Analysis of Volatile Compounds

Head space-solid phase microextraction (HS–SPME) was conducted according to Zielińska et al. [30] with slight modifications. Briefly, 100 mg of dry *E. senticosus* fruits were placed in a 15 mL sealed vial and extracted using a fiber coated with 50/30 µm divinylbenzene–carboxen–polydimethylsiloxane (DVB/CAR/PDMS; Supelco, Bellefonte, PE, USA). The 2-undecanone–2 mg/mL in water (Merck, Poland) was used as an internal standard. Equilibration was performed at 60 °C for 15 min, the fiber exposition time was 15 min, and the thermal desorption was 3 min at 250 °C directly in the gas chromatography (GC) injection port. All analyses were performed in triplicate. The gas chromatography (GC) analysis was performed using Agilent 7890B GC coupled with a 7000GC/TQ system mass spectrometer (Agilent Technologies, Paolo Alto, CA, USA). Separation was carried out on an HP-5 MS column; 30m × 0.25 mm × 0.25 µm (J&W, Agilent Technologies, Palo Alto, CA, USA) at a constant helium flow of 1 mL/min. The injector temperature was set at 250 °C and the sample was applied in a split mode (70:1). The temperature program was 50 °C for 1 min, followed by 5 °C/min to 120 °C, 8 °C/min to 200 °C, then to 250 °C in 16 °C/min and held isothermal for 2 min. The MS source, transfer line, and quadrupole temperature were set at 230 °C, 320 °C, and 150 °C, respectively. The mass spectra were collected in a scan mode from *m/z* 30–400 and the ionization voltage was 70 eV. Data acquisition was performed using Agilent MassHunter Workstation software (version B.08.00). The identification of the compounds was based on a comparison of fragmentation patterns with the NIST17 mass spectra library and they were matched with retention index (RI) obtained by calculation relative to the n-alkane standard (C8–C20; Merc, Poland). The quantitative analysis (expressed as percentages of each compound) was carried out by peak area normalization measurements without a correction factor.

### 3.7. Isolation of Oil and Preparation of Samples

Air-dried and pulverized fruits (5 g) were extracted four times with hexane (4 × 30 mL) using an ultra-sonic bath (4 × 15 min.) The extracts were combined and evaporated in a rotary evaporator. The oil (50 mg) was placed in a volumetric flask (5 mL) and dissolved in 2-propanol. The solution was filtered through a 0.25 µm polyamide membrane filter before HPLC analysis. The oil (10 mg) was dissolved in 500 μL of TBME and derivatized by the addition of 250 μL of TMSH. The whole sample was shaken vigorously, and the GC–MS analysis was performed.

#### 3.7.1. HPLC–PDA Analysis of Ursolic Acid and Tocopherols in the Oil

The analysis was performed on a VWR Hitachi Chromaster 600 chromatograph with a 5430 Diode Array Detector, a 5440 FL Detector, and EZChrom Elite software (Merck, Darmstadt, Germany). A RP18e LiChrospher 100 column (Merck, Darmstadt, Germany) (25 cm × 4.0 mm i.d., 5 µm particle size) was used for the analyses. The identity of compounds was established by comparison of retention times and PDA spectra with the corresponding standards. Ursolic acid was determined using a previously published methodology [31]. An isocratic system was used with the basic chromatographic conditions: the mobile phase consisted of acetonitrile, water, and a 1% aqueous phosphoric acid solution (75:25:0.5 *v/v/v*); eluent flow rate 1.0 mL/min; column temperature 10 °C. The injection volume was 10 μL. The analysis was based on chromatograms recorded with the PDA detector. Data were collected between 200 and 400 nm. The quantitative analysis was performed at λ = 200 nm.

Tocopherols were determined using an isocratic system. The mobile phase consisted of acetonitrile and methanol (5:95 *v/v*). The eluent flow rate was 1.2 mL/min. The column temperature was set at 30 °C. The injection volume was 5 μL. The quantitative analysis was performed using a fluorescence detector with an excitation wavelength at λ = 296 nm and an emission wavelength at λ = 330 nm.

#### 3.7.2. GC–MS Analysis of Fatty Acids in the Oil

The analysis was performed using an Agilent GC–MSD system (GC/MSD 6890N/5975) equipped with a HP-88 Agilent capillary column (60 m × 0.25 mm; 0.20 μm film thickness), MSD ChemStation ver. E.02.02.1431 software (Agilent Technologies, Santa Clara, CA, USA), and a split–splitless injector. The oven temperature was programmed from 110 °C to 190 °C with 8 °C/min, held for 2 min at 110 °C and 13 min at 190 °C. The temperature of the injector was 250 °C. The injection volume was 1 μL (split ratio 150:1; split flow 180 mL/min). Helium was used as a carrier gas at a flow rate of 1.2 mL/min. A quadrupole mass spectrometer with electron ionization (EI) at 70 eV and with a full scan type acquisition mode (50 *m/z* to 500 *m/z*) was used as a detector connected with the GC. The temperature of the MS source and the MS quadrupole was set at 230 °C and 150 °C, respectively. Identification of the constituents was based on a comparison of their mass spectra with the mass spectra library NIST resources and retention times with standards.

### 3.8. Statistical Analysis

Determinations were performed in triplicate. The data were subjected to statistical analysis using Statistica 7.0. (StatSoft, Cracow, Poland). The evaluations were analyzed for one-way analysis of variance. Statistical differences between the treatment groups were estimated by Spearman’s (R) and Person’s (r) test. All statistical tests were carried out at a significance level of α = 0.05.

## 4. Conclusions

The practical aspect of the present results may be the application of the fruits as an ingredient of plant-based products used to treat immune-related diseases, as confirmed by Graczyk et al. [8]. With their chlorogenic acid content, the fruit extracts can be examined as a skin-whitening agent acting as tyrosinase inhibitor and possibly used in the cosmetic industry. In addition to this, we did not find information on doses used in ethnomedicine, showing the need of re-confirmation of the fruits’ activity with regard to therapeutically active doses. Based on these results, the fruits may be a substitution for the roots to prevent exploitation of this endangered plant in some countries. This study has clearly shown that the species can be cultivated in Europe, producing biologically active metabolites. 

## Figures and Tables

**Table 1:** Contents of phenolic acids (mg/g extract ±SD), *n* = 3 (3-CQA—chlorogenic acid, 4-CQA—cryptochlorogenic acid, 5-CQA—neochlorogenic acid, PA—protocatechuic acid. Additional information on HPLC–DAD data is given in the supporting information part).

Sample	3-CQA	4-CQA	5-CQA	PA	Total Content
Fruit	1.08 ± 0.96	0.07 ± 0.01	0.030 ± 0.01	0.08 ± 0.07	1.26
Pericarp	0.66 ± 0.2	0.03 ± 0.02	0.01 ± 0.001	0.04 ± 0.03	0.74
Seed	0.076 ± 0.03	0.008 ± 0.001	0.004 ± 0.002	0.008 ± 0.001	0.096

**Table 2:** Percentage of volatile compounds identified by HS–SPME/GC–MS in *E. senticosus* fruits (% ± SD) (RT—retention time, RI calc—retention index, calculated, RI lit—retention index, literature. Internal standard—2-undecanone. All analyses were performed in triplicate).

No.	RI Lit.	RI Calc.	RT (Min)	Compound Name	Formula	Compound *m/z*	Match Factor (%)	Content
1	930	926	6.01	α-Thujene	C_10_H_16_	93; 91; 77	94.55	0.05 ± 0.02
2	939	933	6.18	α-Pinene	C_10_H_16_	93; 91; 77	95.00	0.12 ± 0.09
3	975	973	7.18	(Z)-Sabinene	C_10_H_16_	93; 91; 77	97.84	2.46 ± 1.19
4	985	987	7.52	6-methyl-5-hepten-2-one	C_8_H_14_O	43; 108; 69; 55	97.50	0.15 ± 0.03
5	990	991	7.63	β-Myrcene	C_10_H_16_	93; 69; 41	96.20	1.43 ± 0.44
6	1002	1005	7.99	α-Phellandrene	C_10_H_16_	93; 77; 136	96.90	2.22 ± 0.6
7	1011	1010	8.15	3-Carene	C_10_H_16_	93; 79; 121	97.90	0.37 ± 0.012
8	1026	1025	8.54	*O*-Cymene	C_10_H_14_	119; 134; 91	97.90	1.40 ± 0.26
9	1029	1029	8.65	Limonene	C_10_H_16_	93; 68; 79	98.50	2.81 ± 0.54
10	1037	1038	8.91	(Z)-β-Ocimene	C_10_H_16_	93; 91; 79	93.24	0.05 ± 0.01
11	1050	1048	9.19	(E)-β-Ocimene	C_10_H_16_	93; 91; 79	97.10	0.86 ± 0.15
12	1059	1059	9.49	γ-Terpinene	C_10_H_16_	93; 136; 77	98.60	0.11 ± 0.01
13	1070	1067	9.73	(Z)-Sabinene hydrate	C_10_H_18_O	71; 93; 43	98.50	0.90 ± 0.09
14	1088	1089	10.33	Terpinolene	C_10_H_16_	121; 93; 136	95.04	0.16 ± 0.01
15	1159	1159	12.30	Sabina ketone	C_9_H_14_O	81; 96; 67; 55	83.10	0.08 ± 0.01
16	1177	1179	12.85	Terpinen-4-ol	C_10_H_18_O	71; 111; 93	96.52	0.15 ± 0.02
17	1294	1295	15.99	2-Undecanone (IS)	C_11_H_22_O	58; 43; 71	93.20	3.79 ± 6.31
18	1294	1299	16.11	Methyl myrtenate	C_11_H_16_O_2_	105; 137; 91;77	94.50	0.07 ± 0.02
19	1376	1381	17.91	α-Copaene	C_15_H_24_	161;119; 105; 93	98.20	1.05 ± 0.16
20	1388	1390	18.11	β-Bourbonene	C_15_H_24_	81; 123; 161	94.90	0.12 ± 0.02
21	1408	1409	18.49	7-epi-Sesquithujene	C_15_H_24_	119; 93; 91; 69	93.00	0.14 ± 0.02
22	1419	1426	18.81	β-Caryophyllene	C_15_H_24_	93; 91; 133; 79	97.90	0.91 ± 0.14
23	1432	1436	18.99	(Z)-β-Copaene	C_15_H_24_	161; 105; 91; 119	99.00	0.25 ± 0.04
24	1434	1441	19.08	(E)-α-Bergamotene	C_15_H_24_	119; 93; 91; 69	97.60	0.54 ± 0.07
25	1456	1463	19.49	(E)-β-Farnesene	C_15_H_24_	69; 93; 133; 161	90.00	19.46 ± 0.78
26	1466	1471	19.64	(Z)-Muurola-4,5-diene	C_15_H_24_	161; 105; 91; 204	94.20	0.26 ± 0.04
27	1481	1490	20.00	Germacrene d	C_15_H_24_	161; 105; 91; 119	94.60	12.88 ± 1.10
28	1497	1498	20.16	(E,Z)-α-Farnesene	C_15_H_24_	93; 119; 69; 107	92.10	12.84 ± 0.85
29	1511	1515	20.43	(E)-β-Bisabolene	C_15_H_24_	93; 69; 94; 109	95.70	12.64 ± 0.70
30	1513	1522	20.55	γ-Cadinene	C_15_H_24_	161; 93; 105; 119	97.70	0.55 ± 0.06
31	1522	1530	20.68	β-Sesquiphellandrene	C_15_H_24_	69; 161; 93; 91	95.70	2.6 ± 0.22
32	1532	1538	20.81	(E)-γ-Bisabolene	C_15_H_24_	93; 107; 135; 119	94.60	3.26 ± 0.43
33	1540	1548	20.97	(E)-α-Bisabolene	C_15_H_24_	93; 119; 121; 80	95.70	1.63 ± 0.20
34	1563	1559	21.15	(E)-Nerolidol	C_15_H_26_O	69; 93; 81; 83	85.30	0.23 ± 0.06
35	1607	1619	22.12	β-Oplopenone	C_15_H_24_O	177; 43; 93; 79	93.40	0.15 ± 0.02
36	1658	1665	22.80	α-Bisabolol oxide B	C_15_H_26_O_2_	143; 105; 85; 81	97.66	0.76 ± 0.12
37	1685	1692	23.20	α-Bisabolol	C_15_H_26_O	109; 119; 69; 43	98.90	6.35 ± 0.9
38	2104	2107	27.78	6-Octadecenoic acid, methyl ester, (Z)-	C_19_H_36_O_2_	55; 74; 84; 96	94.52	0.13 ± 0.10
	Monoterpene hydrocarbons and oxygenated monoterpenes							13.17
	Sesquiterpene hydrocarbons and oxygenated sesquiterpenes							76.62
	Other compounds							4.07
	Total							93.86

**Table 3:** Content of fatty acids (% *w/w* ±SD) in *Eleutherococcus senticosus* seed oil (*n* = 3) (RT—retention time, FAME—fatty acid methyl esters, FA—fatty acid, M—molecular ion), LOD—limit of detection, LOQ—limit of quantification.

Compound Name	FA Abbreviation	RT FAME (Min.)	Mass Data FAME	LOD (% *w/w*)	LOQ (% *w/w*)	Content of FA
Palmitic acid	C16:0	12.94 ± 0.1	270 (M),239,227,213,185,143,129,11197,87,74,69,55	0.044	0.132	2.18 ± 0.04
Stearic acid	C18:0	15.08 ± 0.1	298 (M),267,255,241,227,213,199,185,171,157,143,129,115,97,87,83,74,69,55	0.026	0.078	0.29 ± 0.00
Oleic acid	C18:1 (*n-9*)	15.77 ± 0.1	296 (M), 278,264,235,222,180,166,137,123,110,97,83,69,55	0.037	0.111	5.36 ± 0.05
Linoleic acid	C18:2 (*n-6*)	16.95 ± 0.2	294 (M),263,233,220,205,191,178,164,150,135,123,109,95,81,67,59,55	0.013	0.039	18.24 ± 0.26
α-Linolenic acid	C18:3 (*n-3*)	18.56 ± 0.2	292 (M),261,236,193,163,149,121,108,95,79,67,55	0.015	0.045	0.40 ± 0.04
Total						26.47

**Table 4:** Content of selected biologically active compounds (mg/g ± SD) in *Eleutherococcus senticosus* seed oil (*n* = 3) (RT—retention time, LOD—limit of detection, LOQ—limit of quantification. Tocopherol content).

Compound Name	RT (Min.)	Theoretical Plates	Linear Regression Equation	Concentration Range (µg/mL)	Correlation Coefficient (r)	LOD	LOQ	Content
**Fluorescence Detection**						**(ng/mL)**	**(ng/mL)**	
α-Tocopherol	8.36 ± 0.1	6158	y = 1851639x + 17320	0.48–7.20	0.9979	12.0	38.0	0.29 ± 0.00
β- + γ-Tocopherol	7.25 ± 0.1	5779	y = 4739064x + 115013	0.59–8.88	0.9997	6.0	20.0	1.36 ± 0.07
δ-Tocopherol	6.30 ± 0.1	5527	y = 4041480x + 112005	0.55–8.34	0.9999	5.5	18.5	0.33 ± 0.00
**Spectrophotometric Detection**						**(µg/mL)**	**(µg/mL)**	
Ursolic acid	31.4 ± 0.2	6111	y = 30275.9x + 93277	39.20–117.60	0.9989	0.17	0.51	35.72 ± 0.82

## Data Availability

Not applicable.

## Supplementary Materials

The following are available online, Figure S1: Mass spectra for standards (based on the NIST database) and investigated fatty acids. Figure S2: Mass spectra of HS-SPME/GC-MS investigated compounds. Table S1: Chromatographic parameters and calibration data for quantification of investigated eleutherosides and phenolic acids.

## Sample Availability

Samples of the compounds are not available from the authors.
