# Supplementary material for: Pharmacognostic Evaluation and HPLC–PDA and HS–SPME/GC–MS Metabolomic Profiling of Eleutherococcus senticosus Fruits
Source: Molecules. 2021 Mar 31;26(7):1969. doi: 10.3390/molecules26071969 (PMC8036712; doi:10.3390/molecules26071969)
Supplement: Supplementary file 1 [file molecules-26-01969-s001.pdf]

Supplementary Materials

Pharmacognostic Evaluation and HPLC–PDA and HS–SPME/GC–MS Metabolomic Profiling of *Eleutherococcus senticosus* Fruits

Filip Graczyk [1,*], Maciej Strzemski [2], Maciej Balcerek [1], Weronika Kozłowska [3], Barbara Mazurek [4], Michał Karakuła [2], Ireneusz Sowa [2], Aneta A. Ptaszyńska [5] and Daniel Załuski [1]

[1] Department of Pharmaceutical Botany and Pharmacognosy, Ludwik Rydygier Collegium Medicum, Nicolaus Copernicus University, Marie Curie-Skłodowska 9, 85-094 Bydgoszcz, Poland; balcerek@cm.umk.pl (M.B.); daniel_zaluski@onet.eu (D.Z.)

[2] Department of Analytical Chemistry, Medical University of Lublin, Chodźki 4a, 20-093 Lublin, Poland; maciej.strzemski@poczta.onet.pl (M.S.); michal.karakula@umlub.pl (M.K.); i.sowa@umlub.pl (I.S.)

[3] Department of Pharmaceutical Biology, Wroclaw Medical University, Borowska 211, 50-556 Wroclaw, Poland; weronika.kozlowska@umed.wroc.pl

[4] Analytical Department, New Chemical Syntheses Institute, Aleja Tysiąclecia Państwa Polskiego 13a, 24-110 Puławy, Poland; barbara.mazurek@ins.lukasiewicz.gov.pl

[5] Department of Immunobiology, Institute of Biological Sciences, Faculty of Biology and Biotechnology, Maria Curie-Skłodowska University, Akademicka 19 Str., 20-033 Lublin, Poland; anetaptas@wp.pl

* Correspondence: filip.graczk@gmail.com; Tel.: +48-795672587

Abstract: *Eleutherococcus senticosus* (Rupr. et Maxim.) Maxim. is a medicinal plant used in Traditional Chinese Medicine (TCM) for thousands of years. However, due to the overexploitation, this species is considered to be endangered and is included in the Red List, e.g., in the Republic of Korea. Therefore, a new source of this important plant in Europe is needed. The aim of this study was to develop pharmacognostic and phytochemical parameters of the fruits. The content of polyphenols (eleutherosides B, E, E1) and phenolic acids in the different parts of the fruits, as well as tocopherols, fatty acids in the oil, and volatile constituents were studied by the means of chromatographic techniques [HPLC with Photodiode-Array Detection (PDA), headspace solid-phase microextraction coupled to gas chromatography-mass spectrometry (HS–SPME/GC–MS)]. To the best of our knowledge, no information is available on the content of eleutherosides and phenolic acids in the pericarp and seeds. The highest sum of eleutheroside B and E was detected in the whole fruits (1.4 mg/g), next in the pericarp (1.23 mg/g) and the seeds (0.85 mg/g). Amongst chlorogenic acid derivatives (3-CQA, 4-CQA, 5-CQA), 3-CQA was predominant in the whole fruits (1.08 mg/g), next in the pericarp (0.66 mg/g), and the seeds (0.076 mg/g). The oil was rich in linoleic acid (C18:3 (n-3), 18.24%), ursolic acid (35.72 mg/g), and α-tocopherol (8.36 mg/g). The presence of druses and yellow oil droplets in the inner zone of the mesocarp and chromoplasts in the outer zone can be used as anatomical markers. These studies provide a phytochemical proof for accumulation of polyphenols mainly in the pericarp, and these structures may be taken into consideration as their source subjected to extraction to obtain polyphenol-rich extracts.

Keywords: *Eleutherococcus senticosus*; fruits; eleutherosides; nutri-pharmacological; metabolomics; herbs

Table S1. Chromatographic parameters and calibration data for quantification of investigated eleutherosides and phenolic acids.

Compound	Retention time (min.)	Theoretical plates	Linear regression equation	Concentration range (µg/mL)	Correlation coefficient (r)	LOD (µg/mL)	LOQ (µg/mL)
Eleutheroside B	10.55 ± 0.02	5369	y=133974820x + 6183	0.53-8.00	0.9991	0.35	1.05
Eleutheroside E	20.42 ± 0.02	126464	y=498433022x - 104028	0.53-8.00	0.9934	0.97	2.94
Eleutheroside E1	26.81 ± 0.02	41330	y=392169263x − 28199	1.20-18.00	0.9998	0.42	1.26
Protocatechuic acid	10.59 ± 0.02	17565	y=266763646x + 17984	0.75-7.50	0.9996	0.33	1.02
Neochlorogenic acid	13.72 ± 0.02	16088	y=220873737x + 14961	0.61-6.10	0.9941	0.34	1.03
Chlorogenic acid	24.27 ± 0.03	69256	y=220896903x − 52187	2.60-26.00	0.9997	0.35	1.07
Cryptochlorogenic acid	24.80 ± 0.03	51230	y= 236829164x + 16 678	0.58-5.80	0.9998	0.35	1.07

LOD and LOQ were calculated as follows: LOD = 3.3 d/S and LOQ = 10 d/S where: d=the standard deviation of y-intercept of the regression line. S = the average slope of regression lines.

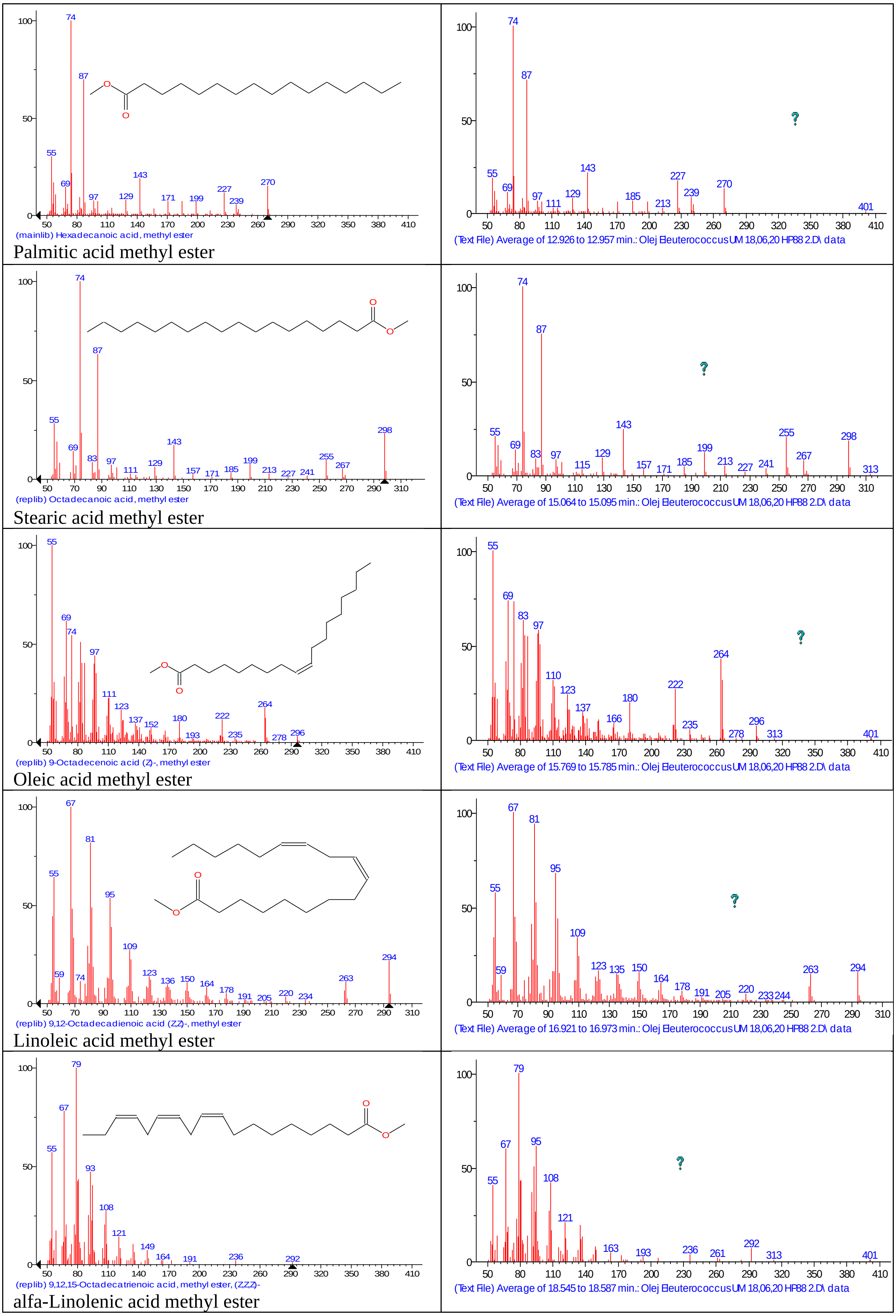

Fig. S1. Mass spectra for standards (based on the NIST database) and investigated fatty acids.

Mass spectra of HS-SPME GC-MS investigated compounds

Library search results	Investigated compounds spectra
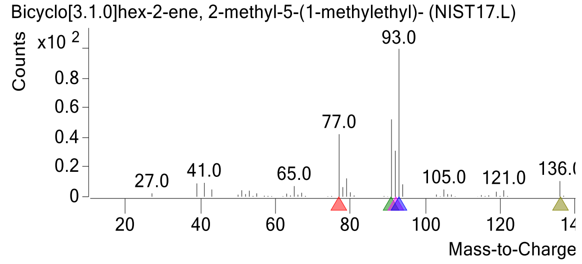	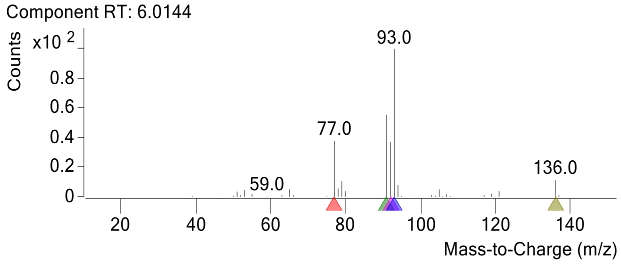

1. α-Thujene

Library search results	Investigated compounds spectra
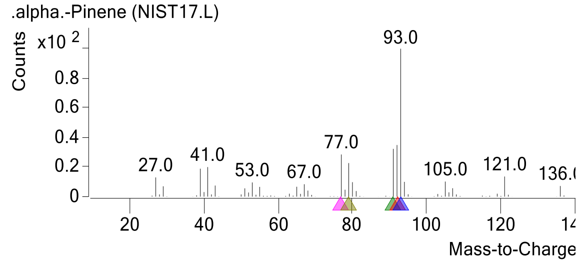	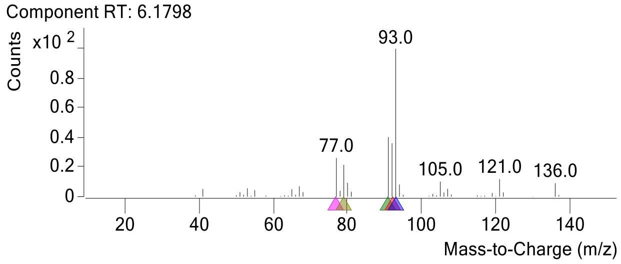

2. α-Pinene

Library search results	Investigated compounds spectra
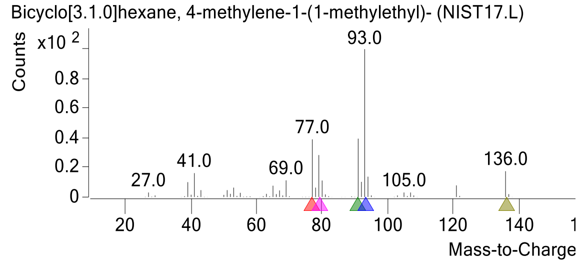	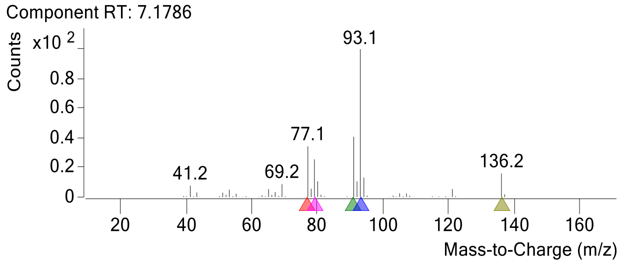

3. (Z)-Sabinene

Library search results	Investigated compounds spectra
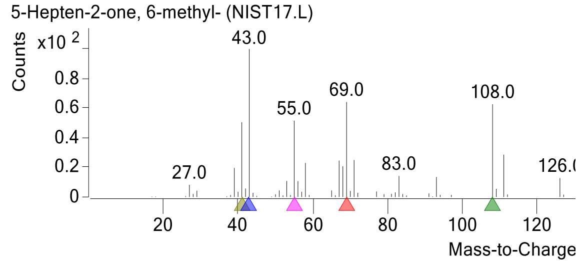	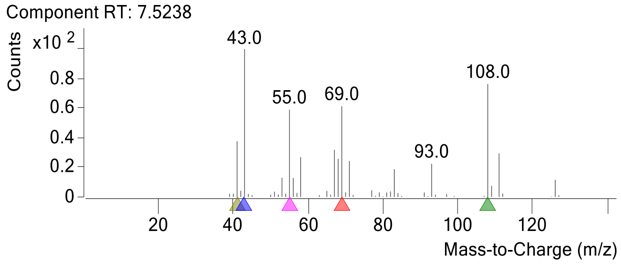

4. 6-methyl-5-hepten-2-one

Library search results	Investigated compounds spectra
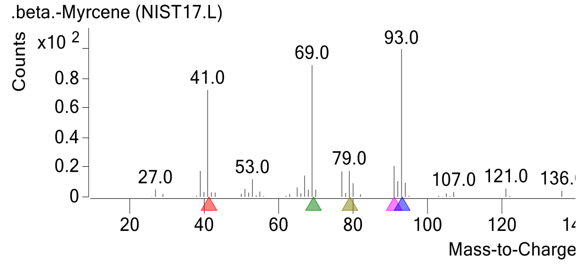	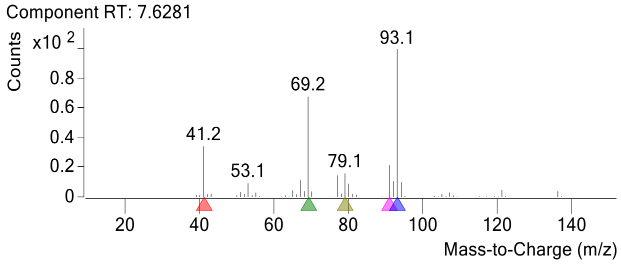

5. β-Myrcene

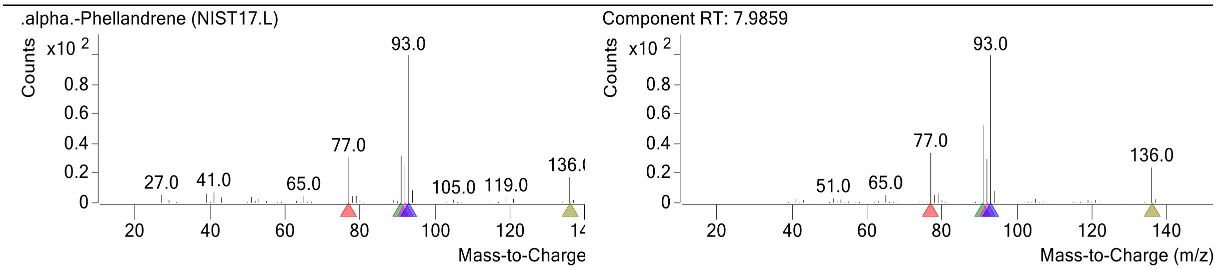

6. α-Phellandrene

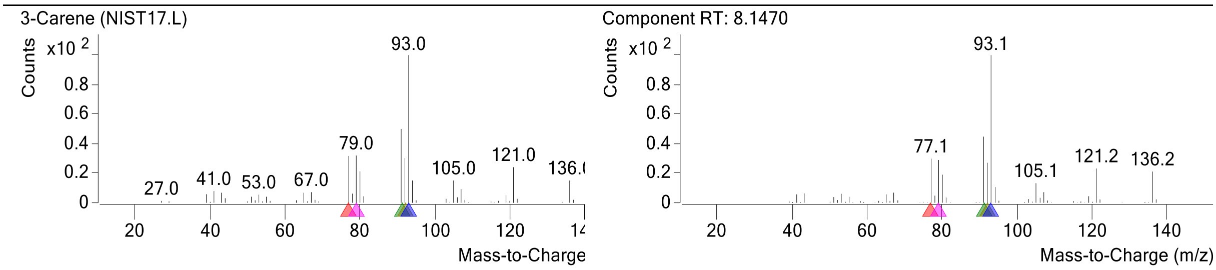

7. 3-Carene

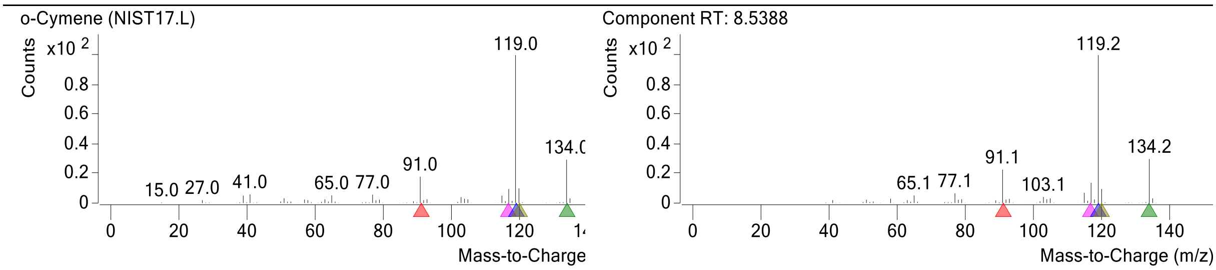

8. o-Cymene

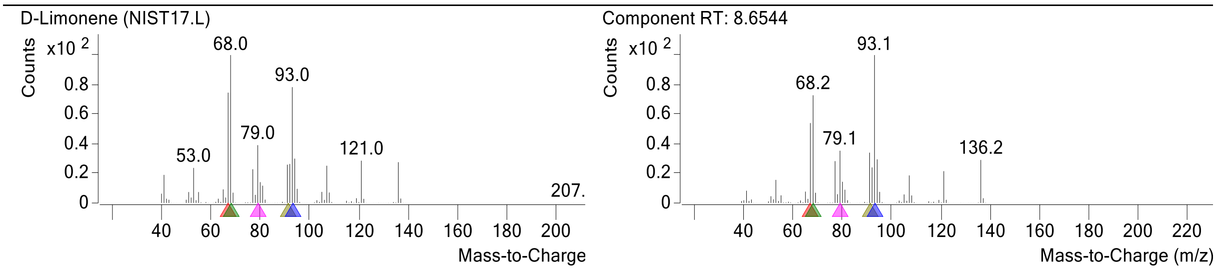

9. Limonene

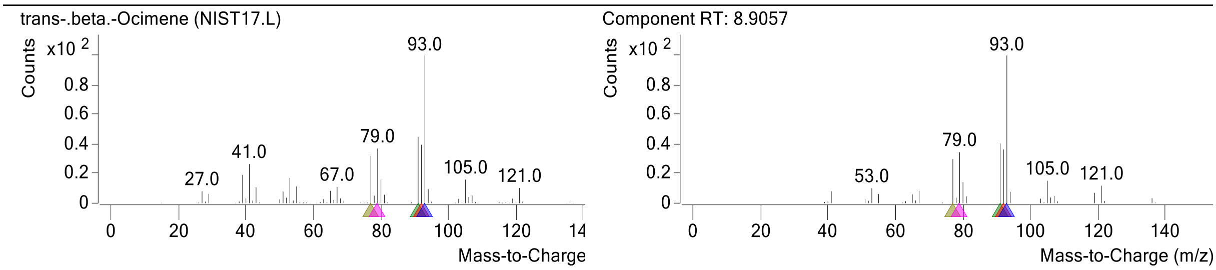

10. (Z)-β-Ocimene

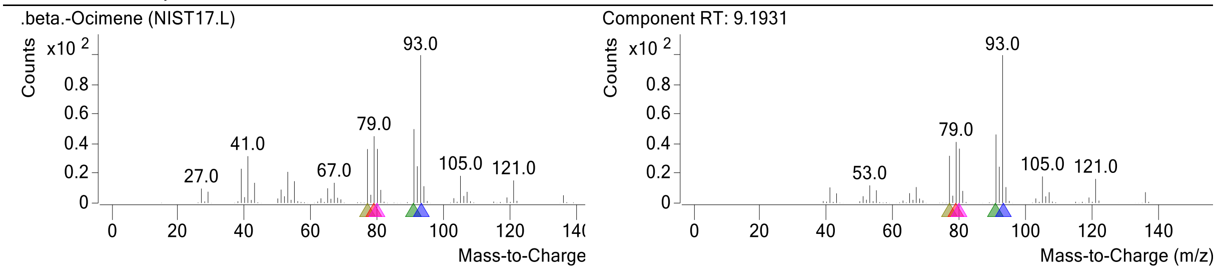

11. (E)-β-Ocimene

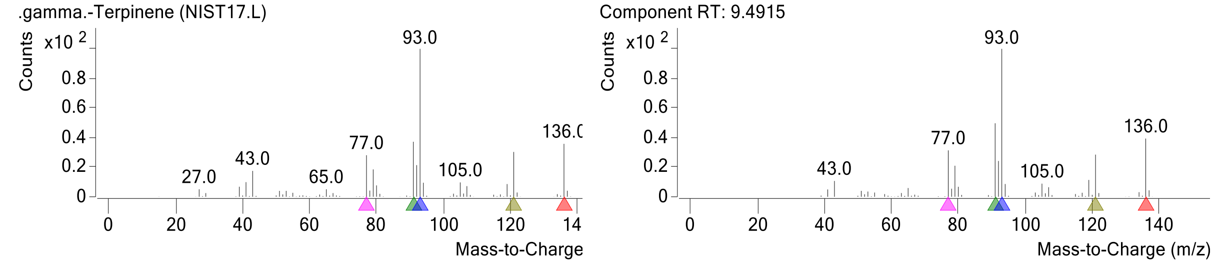

12. γ-Terpinene

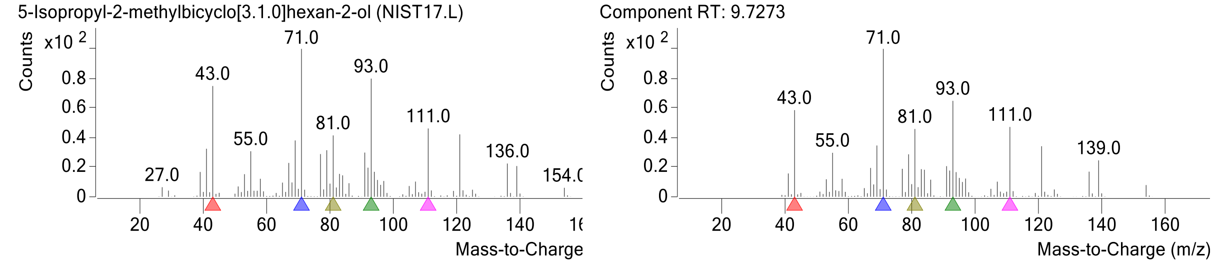

13. (Z)-Sabinene hydrate

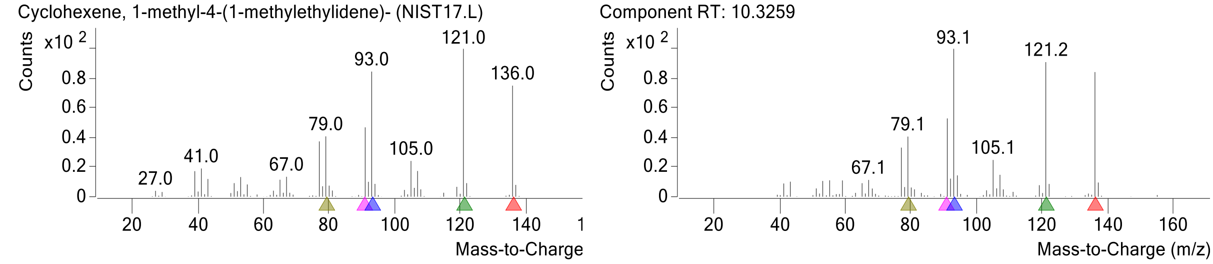

14. Terpinolene

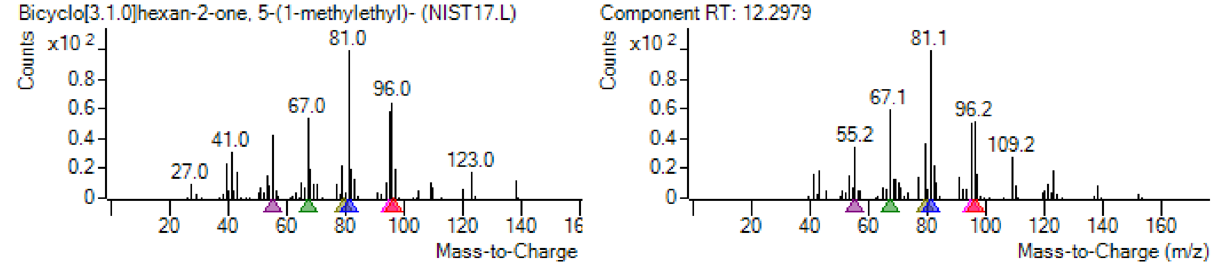

15. Sabina ketone

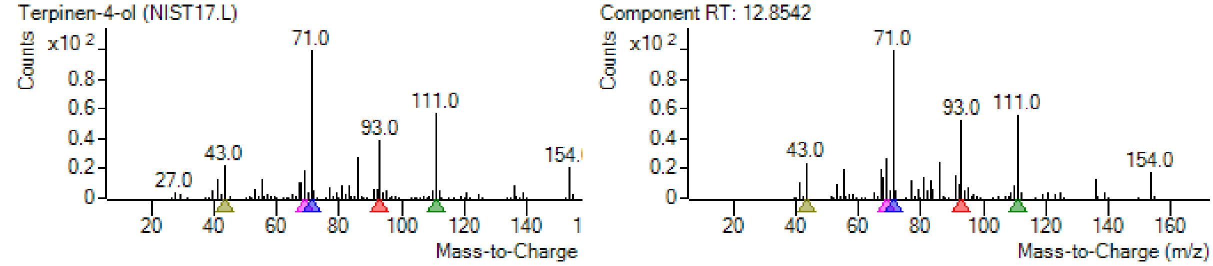

16. Terpinen-4-ol

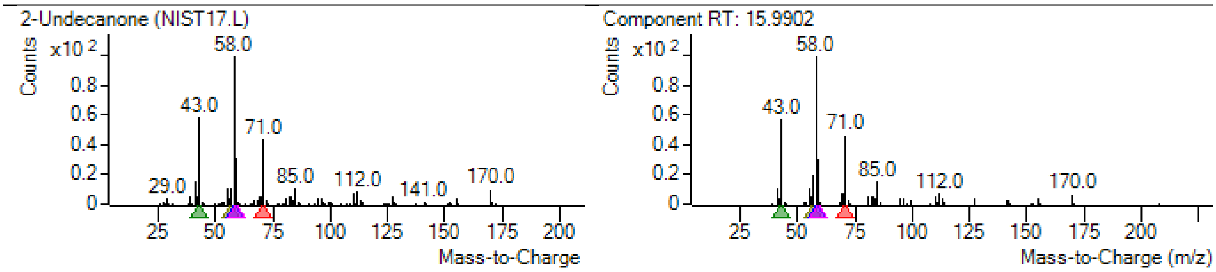

17. 2-Undecanone (IS)

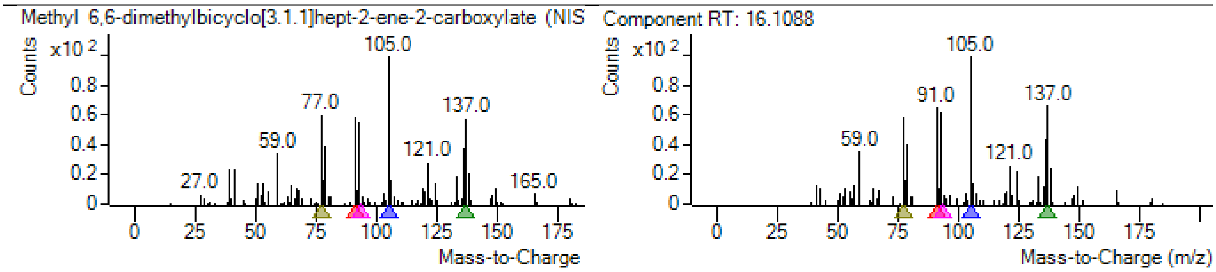

18. Methyl myrtenate

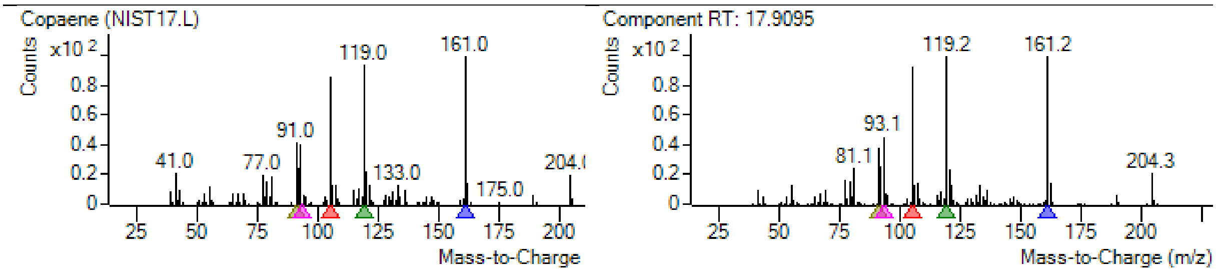

19. α-Copaene

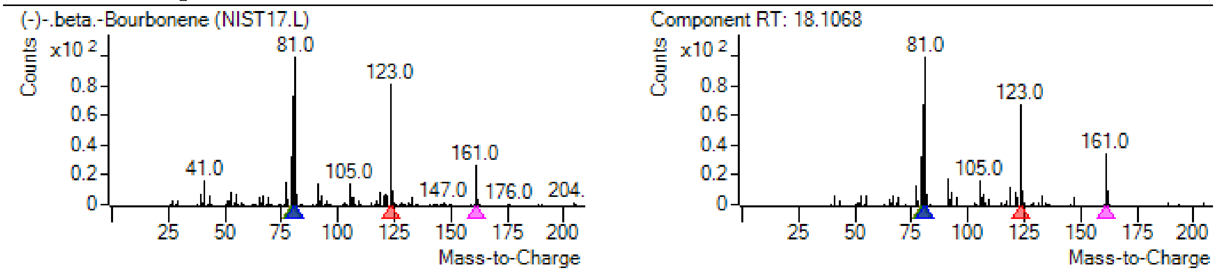

20. β-Bourbonene

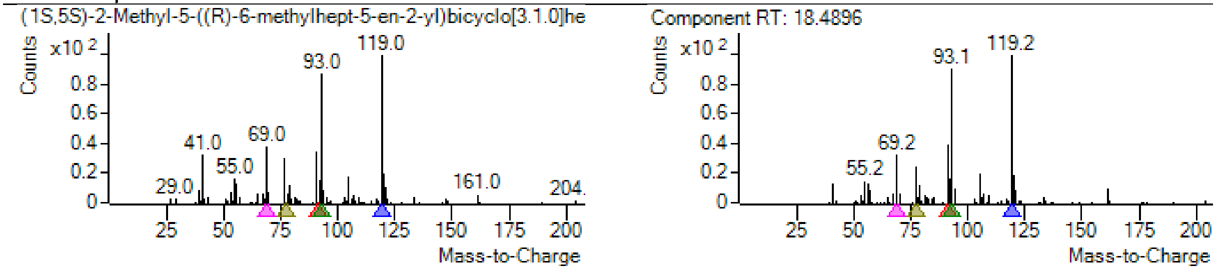

21. 7-epi-Sesquithujene

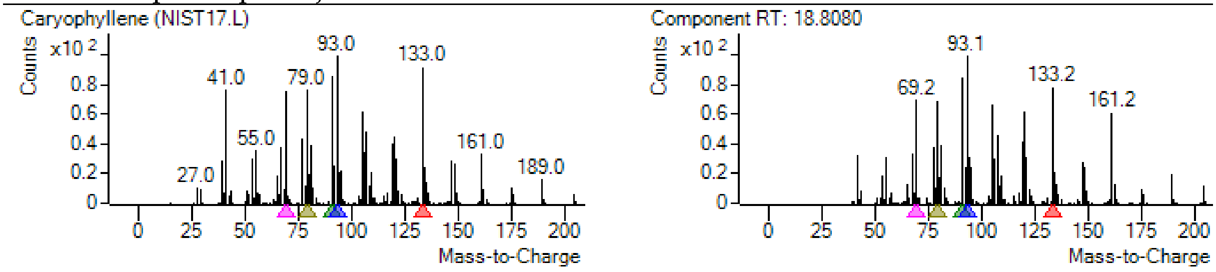

22. β-Caryophyllene

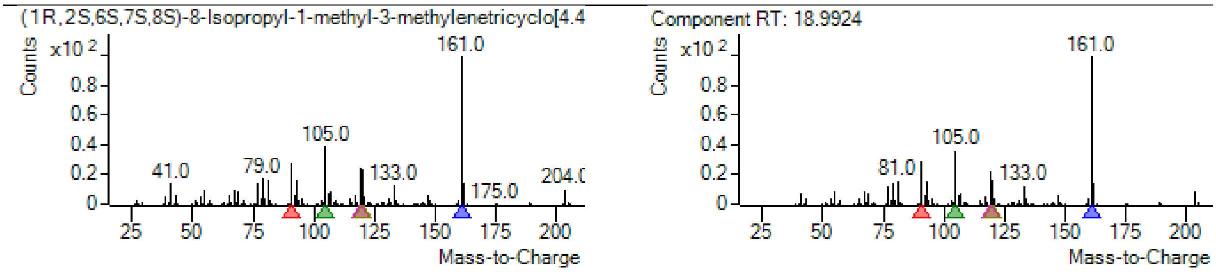

23. (Z)-β-Copaene

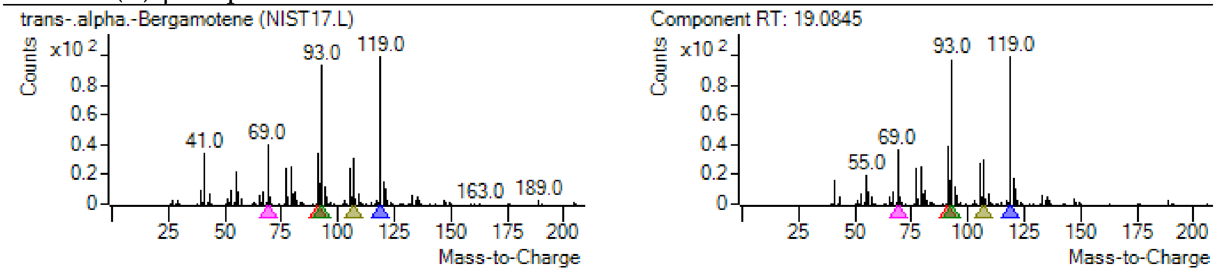

24. (E)-α-Bergamotene

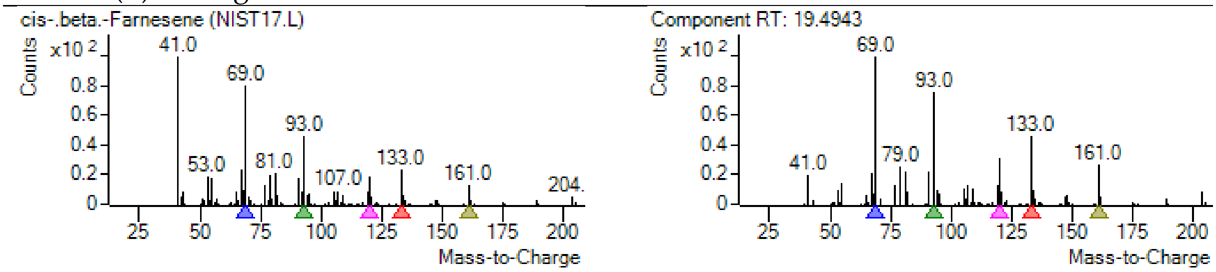

25. (E)-β-Farnesene

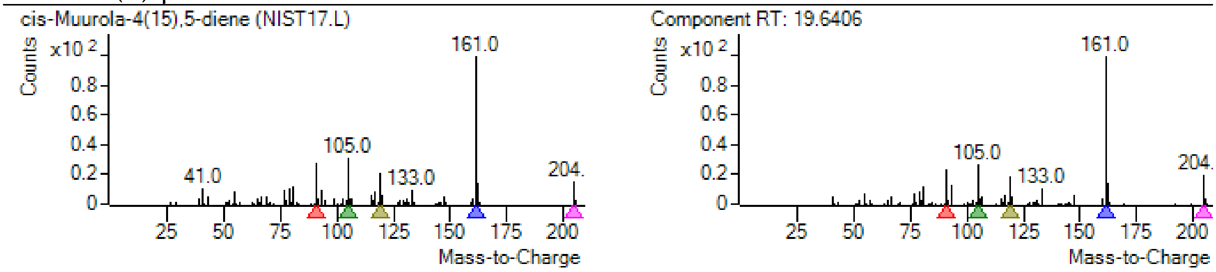

26. (Z)-Muurola-4,5-diene

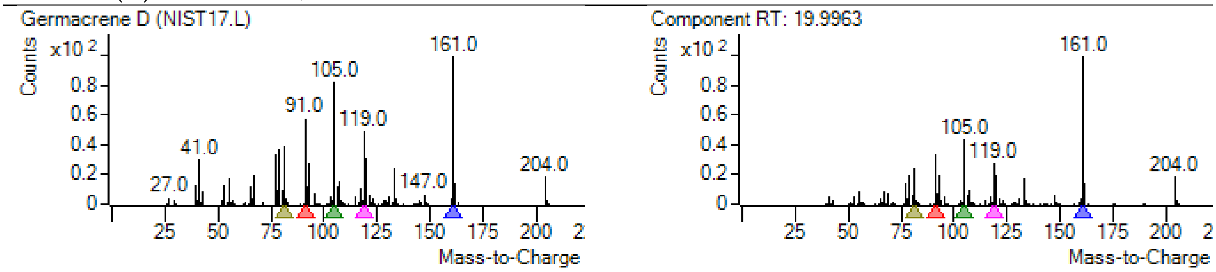

27. Germacrene D

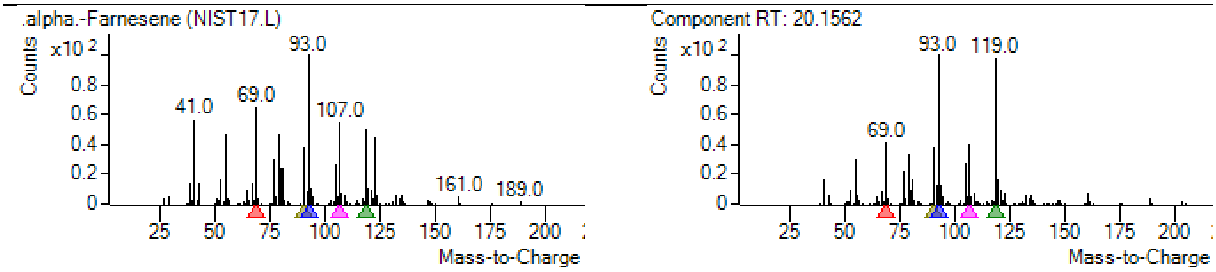

28. (E,Z)-α-Farnesene

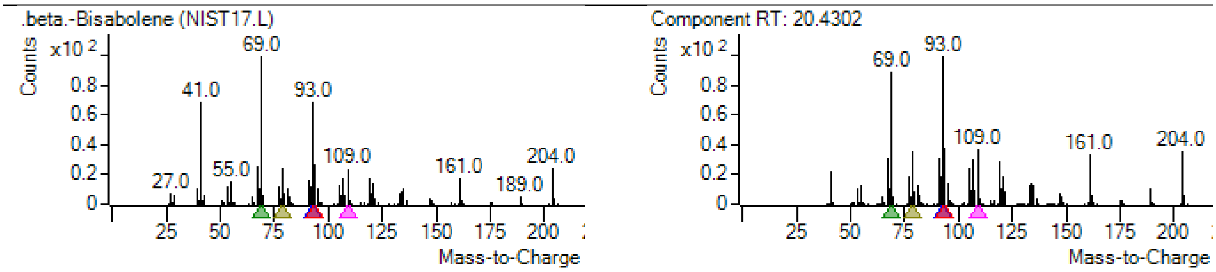

29. (E)-β-Bisabolene

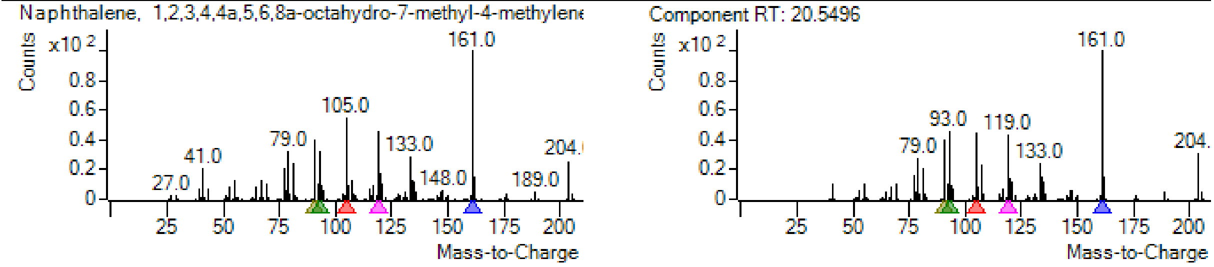

30. γ-Cadinene

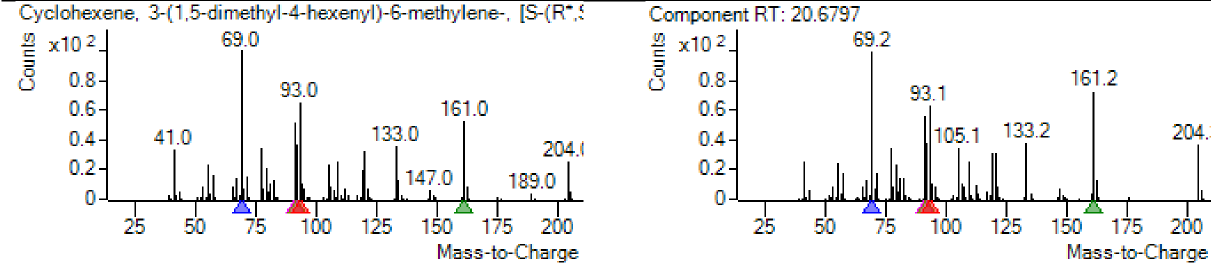

31. β-Sesquiphellandrene

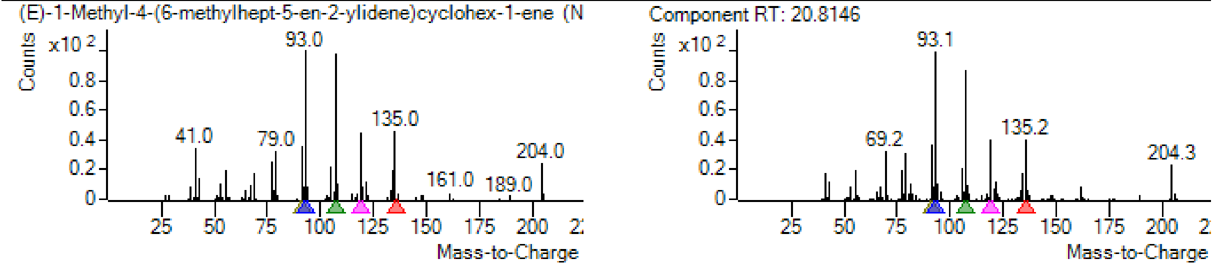

32. (E)-γ-Bisabolene

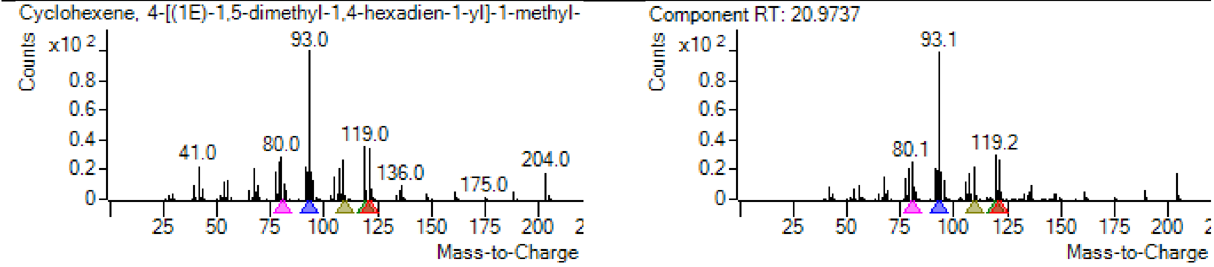

33. (E)-α-Bisabolene

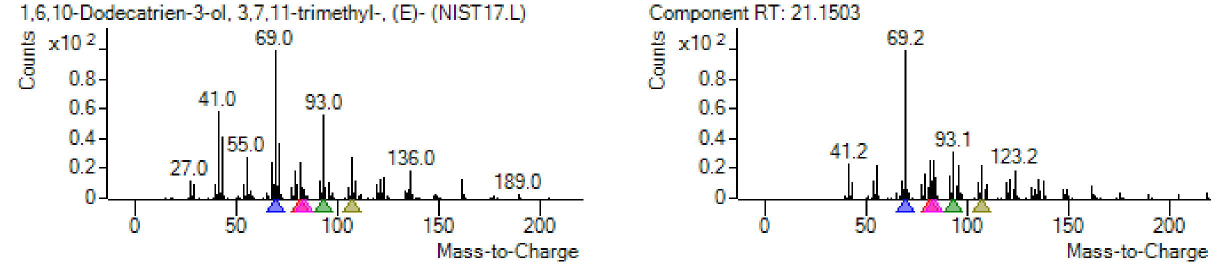

34. (E)-Nerolidol

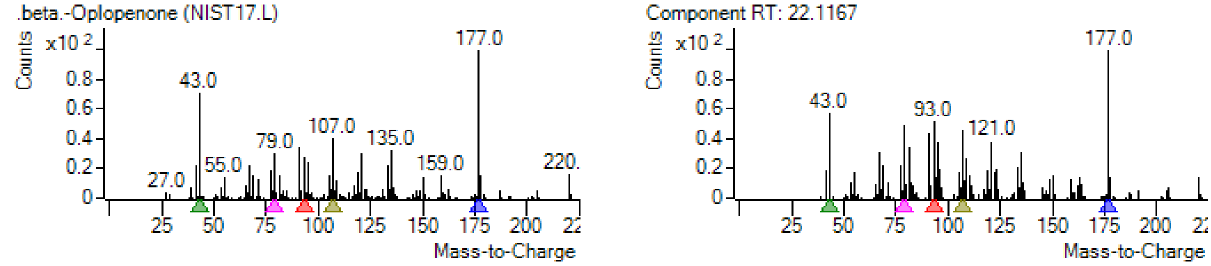

35. β-Oplopenone

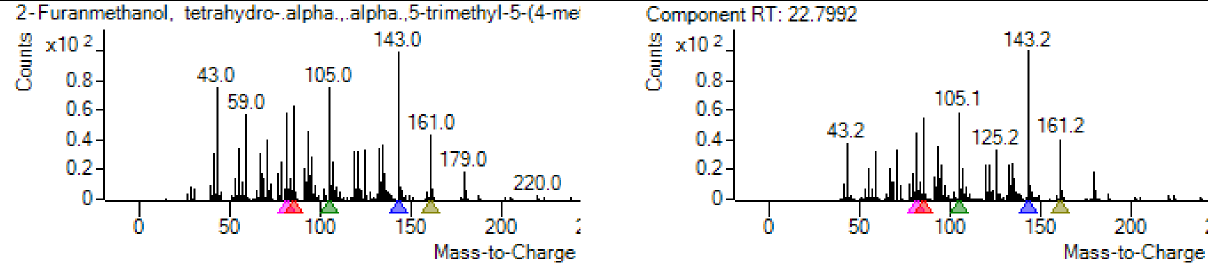

36. α-Bisabolol oxide B

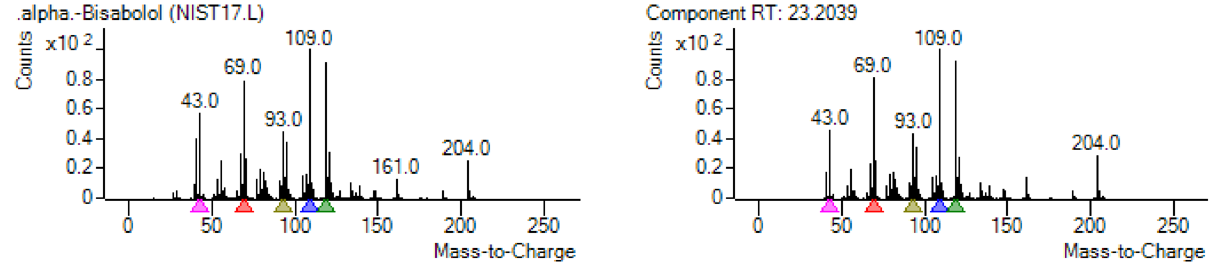

37. α-Bisabolol

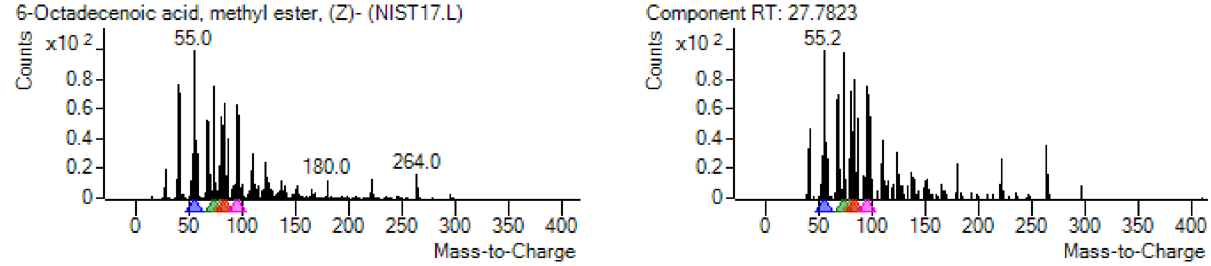

38. 6-Octadecenoic acid, methyl ester, (Z)-

Fig. S2 Mass spectra of HS-SPME/GC-MS investigated compounds.
